# Job burnout and its influencing factors in Chinese medical staffs under China’s prevention and control strategy for the COVID-19 pandemic

**DOI:** 10.1186/s12889-022-14945-5

**Published:** 2023-02-08

**Authors:** Shuzhi Peng, Juhua Zhang, Xingyue Liu, Mengyun Pei, Tingting Wang, Peng Zhang

**Affiliations:** 1 Graduate School, Shanghai University of Medicine & Health Sciences, Shanghai, China; 2 Graduate School, Shanghai University of Traditional Chinese Medicine, Shanghai, China; 3 Department of Integrated Traditional Chinese and Western Medicine, Shanghai University of Medicine and Health Sciences Affiliated Zhoupu Hospital, Shanghai, China; 4 School of Nursing & Health Management, Shanghai University of Medicine & Health Sciences, Shanghai, China; 5 School of Clinical Medicine, Shanghai University of Medicine & Health Sciences, Shanghai, China

**Keywords:** Chinese medical staff, COVID-19, Pandemic prevention and control, Job burnout

## Abstract

**Objective:**

This study aimed to investigate the influencing factors of burnout among grassroots medical staff in China so as to provide a reference for improving their physical, psychological, and social statuses under China's prevention and control strategy for the COVID-19 pandemic and ensuring the sustainable supply of high-quality medical resources.

**Methods:**

This study was performed on medical staff in five primary hospitals in Jiangsu Province, China, from May 1, 2022, to June 1, 2022, using a general information questionnaire and Maslach Burnout Inventory Scale. SPSS 25.0 and Stata 15.0 were used for two-track data entry and analysis. The OLS regression model was established to analyze the influencing factors for the job burnout of health care personnel.

**Results:**

Two hundred seventy valid questionnaires were analyzed. The total score of job burnout was (30.16 ± 10.99). The scores of emotional exhaustion, depersonalization, and self-achievement were (9.88 ± 3.839), (11.99 ± 5.68), and (8.29 ± 5.18), respectively. Feeling depressed and stressed after the pandemic, days working over the past week, and work hours per shift had a positive impact on the Maslach Burnout total score. Increased income and hours working every week had a negative impact on the Maslach Burnout total score. However, sex, age in years, degree, professional title, job category, workplace, marital status, years in practice, health status, active management of health, idea of resignation, and promotion after the pandemic did not affect the Maslach Burnout total score.

**Conclusion:**

The job burnout of medical staff is affected by health conditions, working conditions, the psychological consequences of a pandemic, wages and marital status. Hospital managers should formulate incentive measures according to different psychological changes in medical staff to create a good medical working environment under the normalization of COVID-19 pandemic prevention and control.

## Introduction

It has been more than 2 years since the outbreak of the COVID-19 pandemic in December 2019. Recently, the main strains in China have mutated into the BA.1 and BA.2 strains of Omicron. Covid-19 is highly infectious and may exist for a long time. China will be in the normal stage of pandemic prevention and control for a long time. Regarding the prevention and control measures of China, first, the risk groups are controlled. Positive infected people should be treated thoroughly, and those in close contact should be separated as much as possible. Also, secondary connections should be isolated in a centralized way as far as possible to cut off the chain of pandemic spread in the shortest possible time. Advanced antigen and nucleic acid screening should be performed to identify infected individuals as quickly as possible. Further, community prevention and control should be prioritized. Therefore, besides routine medical treatment, the primary medical staff of China should also undertake a lot of pandemic prevention and control work, leading to medical staff burnout.

The normalization of pandemic prevention and control refers to the need to implement scientific and accurate prevention and control strategies for meeting the urgent needs of the people to restore production and normal lives. The job burnout of medical staff is related to not only their physical and mental health but also the quality of medical services, the life of patients, and even the harmonious and stable development of society. If medical staff are in a state of job burnout, it can affect medical security, resulting in decreased patient satisfaction and eventually deterioration of the doctor–patient relationship.

Job burnout refers to excessive physical and mental consumption and energy failure due to long-term work stress. Maslach believes that job burnout is not caused by occupations or individuals' unilateral causes, but it depends on the balance between personal and professional lives [1]. The higher the balance between personal expectations and professional requirements, the stronger the individual's dedication toward professional activities, and the more infrequent is job burnout. Otherwise, job burnout can occur. Hobfoil and Shirom believe that people are trying to obtain and preserve valuable resources. Job burnout occurs when these valuable resources are lost, or the resources cannot meet the needs [2].

The job burnout of medical staff has become a hot research topic both in China and abroad. Medical institutions and health administrative departments should be aware of the serious consequences of job burnout and take adequate measures to alleviate it. Considering all the surveys on job burnout of medical staff in China, the medical staff in large hospitals are mainly investigated. However, the research on medical staff in small-scale medical institutions (community health service stations, township health centers, village health centers, and so on) is scarce, leading to a lack of comprehensive data [3, 4]. In this study, the medical staff from a city with relatively less economic development and relatively low medical level in northern Jiangsu province were selected to highlight the role of grassroots medical staff in the prevention and control of the pandemic.

Research on job burnout often uses three dimensions: emotional exhaustion, cynicism (or depersonalization), and personal accomplishment (or low efficiency) [5]. Emotional exhaustion refers to the exhaustion of individual emotional resources and related physiological resources, such as extreme fatigue and loss of work enthusiasm [6]. Cynicism or depersonalization refers to an individual who works with a negative or indifferent attitude, displaying irritability, negativity, and lack of emotional investment [7]. Personal accomplishment refers to an individual with inefficiency, lack of a sense of achievement, lack of enthusiasm and motivation, low morale, and nonproductive efforts [8].

Medical staff face continued high pressure, high load, and high-risk working environment, which affect their physical, psychological, and social statuses. Especially in the prevention and control of the COVID-19 pandemic, the medical staff have a lot of job responsibilities. This study investigated the current situation and influencing factors of job burnout among primary medical staff in Jiangsu province, China, to provide a reference for improving their physical, psychological, and social statuses under the normalized pandemic prevention and control and ensuring the sustainable supply of high-quality medical resources.

## Materials and methods

### Participants

This cross-sectional study was conducted from May 1, 2022, to June 1, 2022, by randomly selecting medical staff including doctors and nurses from five primary medical institutions in Jiangsu province, China. The inclusion criteria were as follows: (1) On-the-job medical staff in the hospital; (2) on-the-job duration during the COVID-19 pandemic ≥ 60 days. The exclusion criteria were as follows: (1) healthcare workers who stopped working 6 months or more before the investigation (for example, students studying abroad or suspended from duty); (2) medical staff with obvious mental or organic diseases; and (3) medical staff who refused to participate in the investigation. All participants were aware of the purpose of the survey and signed an agreement authorizing data collection.

### Instruments and measurements

The general information questionnaire was used to investigate the basic characteristics of the participants, such as sex, age, educational background, professional title, health status, increase in income, job promotion, active management of their health, ideas of the resignation and feelings of depression of medical staff after the pandemic. The Maslach Burnout Inventory Scale was used to assess the job burnout of the participants. The scale comprised three dimensions: emotional exhaustion, depersonalization, and low sense of achievement. It had a total of 16 questions and used 5-point Likert scale for scoring: a score of 0 represented "never" and 4 represented "very frequently." The dimension of personal accomplishment was the opposite of emotional exhaustion and depersonalization. The higher the score, the lesser the degree of burnout. The reverse scoring was used to calculate the total score. A higher score indicated more job burnout. The Cronbach's α coefficient for the three dimensions was 0.916, 0.863, and 0.866, respectively. In this study, the overall Cronbach's α coefficient was 0.880 (KMO = 0.871, *P* < 0.001), suggesting that the scale was applicable.

### Sampling method

We used an electronic questionnaire on the questionnaire website. The WeChat link was used to distribute the questionnaire. A unified trained investigator was assigned to each of the five basic-level hospitals randomly selected for the study. The contents of the unified training include: how to judge the questionnaire items, the skills that can be used in the investigation, and some matters needing attention in the investigation process, etc. For those who could not understand the survey questions were described and explained by investigators who have received uniform training. In order to ensure that the investigators describe and explain the contents of the investigation in the same way, all our investigators and interpreters are the same group of uniformly trained personnel. As the participants were medical staff with relatively high education level and understanding ability, no situations requiring explanation by the investigators occurred during the survey. The sample size was determined using sociological research methods and calculated using the following formula: *n* = *p* × *z*^2^ × (1 – *p*)/*e*^2^, where *p* refers to the overall proportion, *z* refers to the confidence coefficient, and *e* refers to the allowable error. If *p* = 0.8, the maximum variance could obtain a relatively conservative sample size. At the same time, the allowable error of 3%–5% and the placing interval of 95% were selected in this survey; the calculated available sample size should be 246–683. Considering the factors such as investigation time, investigators, and the use of funds, the final sample size of 270 was determined.

A total of 300 participants received the questionnaire, and 280 returned the completed questionnaire. The quality control staff checked the questionnaires, and removed that questionnaires with obviously illogical responses or similar answers for most items. The illogical answer refers to some filling out that does not conform to the actual situation, such as working hours > 24 h a day. Similar answer means that all items in a questionnaire chose the first or last option. Details of the 10 deleted questionnaires included: the ages in the two questionnaires are 2 and 5 years old; The three questionnaires were filled in at the working hours of 28 h, 30 h and 27 h per day; There were also 5 questionnaires where the first of all options was selected. Finally, 270 valid questionnaires were retained.

### Statistical analysis

SPSS25.0 and Stata15.0 were used for two-track data entry and data analysis. The scores of job burnout of medical staff were expressed as mean ± standard deviation (x̄ ± s), and *t* test and analysis of variance were used for comparison between the groups. The OLS regression model was established to reveal, compare, and analyze the influencing factors for the job burnout of health care personnel more comprehensively and objectively. The difference was statistically significant with *P* < 0.05.

## Results

Table 1 illustrates the basic situation of the investigated medical staff. The average age of the 270 investigated medical staff was (30.54 ± 6.30) years. The average years in practice were (8.90 ± 6.69) years, and the average working time was (5.69 ± 0.51) days per week. The average working hours were (7.34 ± 1.40) h per day and (41.37 ± 6.29) h per week.

**Table 1:** Characteristics of participants (*n* = 270)

**Variable**	**Value**
Age in year, mean ± SD, (range)	30.54 ± 6.30(18–55)
Sex, *n* (%)	
Female	204(75.56)
Male	66(24.44)
Degree, *n* (%)	
Junior college	54(20.00)
Undergraduate	208(77.04)
Postgraduate and above	8(2.96)
Professional title, *n* (%)	
Primary	90(33.33)
Mid-level	157(58.15)
Senior	23(8.52)
Job category, *n* (%)	
Doctor	31(11.48)
Nurse	239(88.52)
Workplace, *n* (%)	
Country	134(49.63)
Town	136(50.37)
Marital status, *n* (%)	
Married	144(53.33)
Single	126(46.67)
Years in practice, mean ± SD	8.90 ± 6.69
Hours working per week	41.37 ± 6.29
Days working over past week, mean ± SD	5.69 ± 0.51
Hours working per shift, mean ± SD	7.34 ± 1.40

The average Maslach Burnout inventory score of the 270 investigated medical staff was (30.16 ± 10.99), emotional exhaustion subscale score was (9.88 ± 3.84), depersonalization subscale score was (11.99 ± 5.68), and personal accomplishment subscale score was (8.29 ± 5.18). The demographic factors such as sex, educational background, job title, job category, workplace, and marital status were analyzed. A correlation was found between marital status and emotional failure (*P* < 0.05); the occupation burnout score of unmarried personnel was higher than that of married personnel, as shown in Table 2.

**Table 2:** Respondent characteristics

Variable	Value	Maslach Burnout inventory score, mean ± SD			
		Total score	Emotional exhaustion	Depersonalization	Personal accomplishment
Sex, *n* (%)					
Female	204 (75.56)	30.10 ± 10.67	9.81 ± 3.87	11.88 ± 5.59	8.41 ± 5.14
Male	66 (24.44)	30.32 ± 12.02	10.09 ± 3.76	12.33 ± 5.99	7.89 ± 5.33
*t***□**		–0.138	–0.518	–0.560	0.705
Degree, *n* (%)					
Junior college	54 (20.00)	31.28 ± 10.70	10.15 ± 3.98	12.54 ± 5.83	8.59 ± 4.87
Undergraduate	208 (77.04)	29.97 ± 11.07	9.84 ± 3.85	11.88 ± 5.67	8.25 ± 5.25
Postgraduate and above	8 (2.96)	27.38 ± 11.64	9.00 ± 2.67	11.38 ± 5.42	7.00 ± 5.76
*F***□**		0.565	0.351	0.338	0.343
Professional title, *n* (%)					
Primary	90 (33.33)	29.36 ± 10.93	9.59 ± 3.81	11.84 ± 5.82	7.92 ± 5.11
Mid-level	157 (58.15)	31.12 ± 11.01	10.24 ± 3.87	12.41 ± 5.54	8.47 ± 5.23
Senior	23 (8.52)	26.70 ± 10.58	8.57 ± 3.44	9.70 ± 5.69	8.43 ± 5.30
*F***□**		1.998	2.304	2.368	0.330
Job category, *n* (%)					
Doctor	31 (11.48)	29.42 ± 11.88	9.81 ± 4.00	10.97 ± 5.79	8.65 ± 5.23
Nurse	239 (88.52)	30.25 ± 10.89	9.89 ± 3.82	12.13 ± 5.66	8.24 ± 5.18
*t***□**		–0.396	–0.110	–1.068	0.411
Workplace, *n* (%)					
Country	134 (49.63)	29.97 ± 11.29	9.81 ± 3.75	11.81 ± 5.75	8.35 ± 5.30
Town	136 (50.37)	30.34 ± 10.72	9.94 ± 3.94	12.18 ± 5.63	8.22 ± 5.08
*t***□**		–0.275	–0.273	–0.535	0.206
Marital status, *n* (%)					
Married	144 (53.33)	29.57 ± 10.86	9.42 ± 3.67	11.61 ± 5.67	8.54 ± 5.49
Single	126 (46.67)	30.83 ± 11.15	10.40 ± 3.97	12.43 ± 5.68	7.99 ± 4.81
*t***□**		–0.936	–2.124^*^	–1.181	0.869

^*^P < 0.05, ^**^P < 0.01

During the outbreak of the pandemic, health conditions, increase in income, job promotion, and ideas of resignation showed significant differences (*P* < 0.05) in occupational burnout scores, emotional exhaustion, depersonalization, and self-achievement. The active management of their own health and the feeling of depression and stress after the pandemic did not show significant differences in self-achievement (*P* > 0.05). The specific conditions are shown in Table 3.

**Table 3:** Comparison of factors influencing burnout during the pandemic

Variable	*n* (%)	Maslach Burnout inventory score, mean ± SD			
		Total score	Emotional exhaustion	Depersonalization	Personal accomplishment
Health status					
Bad	46 (17.04)	45.13 ± 4.75	14.15 ± 1.98	18.89 ± 2.70	12.09 ± 4.52
Regular	197 (72.96)	29.48 ± 6.85	9.66 ± 3.13	11.59 ± 4.48	8.23 ± 4.77
Good	27 (10.00)	9.59 ± 4.23	4.19 ± 2.37	3.19 ± 1.90	2.22 ± 2.71
*F***□**		272.862^**^	102.873^**^	132.139^**^	39.788^**^
Proactively manage your health					
No	93 (34.44)	32.38 ± 11.36	10.71 ± 3.66	13.03 ± 5.07	8.63 ± 5.15
Yes	177 (65.56)	28.99 ± 10.64	9.44 ± 3.87	11.45 ± 5.92	8.10 ± 5.20
*t***□**		2.428^*^	2.609^**^	2.196^*^	0.802
Increased income					
No	208 (77.04)	33.50 ± 9.25	10.64 ± 3.50	13.45 ± 5.25	9.41 ± 4.86
Yes	62 (22.96)	18.94 ± 8.75	7.31 ± 3.84	7.11 ± 4.14	4.52 ± 4.40
*t***□**		11.018^**^	6.447^**^	9.901^**^	7.100^**^
Promotion					
Unchanged	234 (86.67)	32.36 ± 9.06	10.53 ± 3.40	12.98 ± 5.06	8.85 ± 4.88
Promotion	36 (13.33)	15.83 ± 11.79	5.64 ± 3.86	5.58 ± 5.33	4.61 ± 5.64
*t***□**		8.052^**^	7.886^**^	8.100^**^	4.750^**^
Feeling depressed					
No	102 (37.78)	24.82 ± 11.53	7.85 ± 4.13	9.24 ± 5.64	7.74 ± 5.61
Yes	168 (62.22)	33.39 ± 9.29	11.11 ± 3.07	13.67 ± 5.03	8.62 ± 4.89
*t***□**		–6.358^**^	–6.893^**^	–6.705^**^	-1.361
Resignation idea					
No	119 (44.07)	24.88 ± 10.25	8.12 ± 3.81	8.93 ± 5.05	7.83 ± 5.56
Yes	151 (55.93)	34.31 ± 9.73	11.26 ± 3.26	14.40 ± 4.95	8.64 ± 4.85
*t***□**		–7.723^**^	–7.313^**^	–8.938^**^	–1.278

^*^P < 0.05, ^**^P < 0.01

All these changes happen after the pandemic

The influencing factors investigated in this study were included in the model as independent variables, and OLS regression analysis was performed. The *F* test was performed on the model (*P* < 0.05), indicating that at least one independent variable could affect the four dependent variables (see Table 4 for details).

**Table 4:** OLS regression analysis results

Variable	Maslach burnout total score		Emotional exhaustion		Depersonalization		Personal accomplishment	
	Regression coefficient	95% CI	Regression coefficient	95% CI	Regression coefficient	95% CI	Regression coefficient	95% CI
Constant	-155.131^**^ (-18.002)	-172.021〜-138.242	-42.870^**^ (-6.828)	–55.176〜 –30.563	–78.476^**^ (–10.159)	-93.616–63.336	–33.786^**^ (–3.270)	-54.035〜-13.537
Marital status	0.199 (0.528)	-0.539〜0.936	0.693^*^ (2.022)	0.021〜1.365	0.214 (0.591)	-0.494〜0.921	–0.708 (–1.374)	-1.718〜0.302
Health status	-0.285 (-0.536)	-1.328〜0.757	0.436 (1.237)	–0.255〜1.128	0.663 (1.422)	-0.251〜1.577	–1.385^*^ (-2.161)	-2.640〜-0.129
Increased income after the pandemic	-3.301^**^ (-6.596)	0.660〜2.162	-0.010 (-0.021)	0.677〜2.068	–0.872 (-1.812)	0.133〜1.633	–2.419^**^ (–3.262)	-1.979〜0.289
Feeling depressed and stressed after the pandemic	1.411^**^ (3.682)	-4.282〜-2.320	1.373^**^ (3.869)	-0.975〜0.954	0.883^*^ (2.308)	-1.815〜0.071	–0.845 (–1.461)	-3.873〜-0.966
Resignation idea	0.380 (1.048)	-4.189〜0.604	0.525 (1.523)	-2.819〜0.255	1.417^**^ (3.774)	-3.291〜0.940	–1.562^**^ (–2.885)	-2.252〜3.582
Working hours per week	-1.782^**^ (-10.770)	21.864〜27.195	-0.429^**^ (-4.506)	4.911〜8.377	-0.704^**^ (-5.204)	8.922〜13.785	–0.649^**^ (–3.953)	3.569〜9.495
Days working over the past week	24.529^**^ (18.036)	15.006〜 18.709	6.644^**^ (7.514)	3.033〜5.195	11.353^**^ (9.152)	5.560〜8.487	6.532^**^ (4.321)	3.945〜7.495
Hours working per shift	16.858^**^ (17.845)	-2.106〜 -1.458	4.114^**^ (7.462)	-0.616〜-0.242	7.024^**^ (9.406)	-0.970〜-0.439	5.720^**^ (6.315)	-0.970〜-0.327
*R* ^2^	0.931		0.611		0.763	0.406		
*F* **□**	*F*(17,252) = 257.845 ^**^		*F* (17,252) = 33.689 ^**^		*F* (17,252) = 66.998 ^**^	*F* (17,252) = 19.469 ^**^		
D-W	0.629		1.819		1.524	1.517		

^*^P < 0.05, ^**^P < 0.01. *T* value is in parentheses. Variables that are not statistically significant are not presented

## Discussion

Job burnout has become a common problem among medical staff. Goldberg [9] conducted a survey of emergency physicians and found that 60% of them had moderate-to-high levels of job burnout. Kluger [10] conducted a study on Australian anesthesiologists and found that 20%, 20%, and 36% of anesthesiologists, respectively, suffered from emotional exhaustion, depersonalization, and low personal accomplishment. A survey conducted by the British Medical Association found that 21% of the health care workers surveyed said that they were under excessive pressure that was difficult to cope with, 61% said that the pressure was excessive but tolerable, and 55% of the respondents could not accept the negative impact of work pressure on the quality of life [11].

We found that the total score of job burnout did not show significant differences in sex, educational background, professional title, occupation type, occupation location, and marital status. The study pointed out that men showed a higher level of job burnout than their female counterparts, and the level of job burnout of unmarried people was lower than that of married people [12–15]. However, the results of this study did not find the impact of these factors on job burnout, which might be related to the difference in the selection of our sample size [16]. The dimension of emotional exhaustion in job burnout was compared with the aforementioned factors. A difference was found in marital status and emotional exhaustion, and the job burnout score of unmarried personnel was higher than that of married personnel. This was contrary to the results of previous studies [4, 16, 17], probably because unmarried personnel were full of expectation and enthusiasm for their jobs [18]. When their enthusiasm was not released and met, job burnout was likely to occur. Married people, due to family responsibilities, expend more energy and are satisfied with their work in the current situation [19, 20]. These reasons may lead to a lower level of job burnout.

This study also investigated the impact of health conditions of health care personnel, active management of their health, increased income, job promotion, feeling of depression, and the idea of resignation on job burnout after the outbreak of the pandemic, which was consistent with the results of previous studies [7, 21, 22]. The study also found that the total score of job burnout based on the physical condition was bad > regular > good. Therefore, medical staff should pay attention to maintaining good physical condition at ordinary times, which is conducive to reducing the occurrence of job burnout [23]. The occupational burnout scores of medical staff who did not actively manage their own health were higher than those of medical staff who actively managed their own health, indicating that medical workers should learn to actively manage their own health [24]. The occupational burnout scores of medical staff with increased income were lower than those with unchanged income after the pandemic, indicating that hospital managers should appropriately improve the income of medical staff who have made positive contributions to the prevention and control of the pandemic [25]. The level of job burnout of medical staff with job promotion was lower than that of medical staff without promotion, which also reminded hospital managers to formulate an appropriate incentive mechanism for pandemic prevention and control personnel [26]. The level of job burnout of the medical staff who felt depressed and nervous was higher than that of the medical staff who did not feel the same, indicating that our medical staff should learn to manage their emotions. At the same time, it indicated that hospital managers should pay more attention to the emotional changes in the medical staff and guide their positive emotions [27]. The total score of job burnout of the people who had the idea of resignation was higher than that of the medical staff who did not have the idea of resignation, indicating that hospital managers should always pay attention to the idea of the resignation of medical staff and the reason behind it and formulate corresponding policies and measures [28–31].

The results of the OLS regression analysis showed that the R-square values of the model were 0.931, 0.611, 0.763, and 0.406, implying that the model had more significance in explaining the total score of job burnout. Therefore, our findings were mainly explained by the influence of these independent variables on the total score of job burnout. Job burnout not only affects the physical and mental health, quality of life, and job performance of medical staff, but also may lead to burnout in the whole organization, thus seriously affecting interpersonal relationships, medical quality, and efficiency [32]. Based on the results of this survey, it was suggested that the families should recognize the occupation of medical workers, give more psychological support to medical staff, and find ways to relieve stress. Also, the hospital management personnel should fully respect the expression of medical staff, improve their salary levels, strengthen the cultivation of organizational skills, improve their working ability, reduce occupational burden, strengthen humanistic care, and encourage medical staff to improve their social support network [33, 34]. The level of job burnout of medical staff is affected by various factors. The hospital management personnel should formulate corresponding intervention measures for the relevant influencing factors to reduce the level of job burnout of medical staff [35]. We also found that the longer the working hours, the higher the score of medical staff's job burnout, which was consistent with previous findings [7]. Therefore, we believed that the working hours were one of the causes of job burnout. Therefore, the hospital managers should pay attention to the working hours when scheduling and reasonably arrange the shifts of medical staff.

At present, several cities and regions in China are gradually exploring the implementation of standardized nucleic acid testing. Capital cities and cities with a population of 10 million are establishing 15-min walking distance for nucleic acid testing. Nucleic acid detection is the gold standard for determining the infection in COVID-19. Normalized nucleic acid detection is carried out in areas with a high risk of pandemic input, which is conducive to improving the sensitivity of pandemic monitoring and early warning and identifying potential risks earlier. At the same time, major cities, according to the actual situation of the local reasonable layout of nucleic acid sampling points, can allow citizens to go to the nearest nucleic acid testing center. The normalization of nucleic acid sampling can reduce the occurrence of cases. Although the current nucleic acid collection work has been handed over to the third-party professional testing institutions, the medical staff is still the main force in the first line of anti-pandemic strategy. Under the current pandemic prevention and control policy in China, the occupational burnout of medical staff deserves our attention.

Our study had some limitations. First, it was a cross-sectional study that could not determine the causal relationship between the factors investigated and job burnout. Second, the sample size of this survey was limited due to time, funding, and other factors, and the population surveyed did not represent the national situation.

## Conclusions

The job burnout of medical staff is affected by health conditions, working conditions, the psychological consequences of a pandemic, wages and marital status. Hospital managers should formulate corresponding incentive measures according to the different psychological changes in medical staff to create a good medical working environment under the normalization of COVID-19 pandemic prevention and control.

## Data Availability

The datasets used and analyzed during the current study available from the corresponding author on reasonable request.
